# Integrating Extrinsic and Intrinsic Cues into a Minimal Model of Lineage Commitment for Hematopoietic Progenitors

**DOI:** 10.1371/journal.pcbi.1000518

**Published:** 2009-09-25

**Authors:** Santhosh Palani, Casim A. Sarkar

**Affiliations:** 1 Department of Bioengineering, University of Pennsylvania, Philadelphia, Pennsylvania, United States of America; 2 Department of Chemical & Biomolecular Engineering, University of Pennsylvania, Philadelphia, Pennsylvania, United States of America; California Institute of Technology, United States of America

## Abstract

Autoregulation of transcription factors and cross-antagonism between lineage-specific transcription factors are a recurrent theme in cell differentiation. An equally prevalent event that is frequently overlooked in lineage commitment models is the upregulation of lineage-specific receptors, often through lineage-specific transcription factors. Here, we use a minimal model that combines cell-extrinsic and cell-intrinsic elements of regulation in order to understand how both instructive and stochastic events can inform cell commitment decisions in hematopoiesis. Our results suggest that cytokine-mediated positive receptor feedback can induce a “switch-like” response to external stimuli during multilineage differentiation by providing robustness to both bipotent and committed states while protecting progenitors from noise-induced differentiation or decommitment. Our model provides support to both the instructive and stochastic theories of commitment: cell fates are ultimately driven by lineage-specific transcription factors, but cytokine signaling can strongly bias lineage commitment by regulating these inherently noisy cell-fate decisions with complex, pertinent behaviors such as ligand-mediated ultrasensitivity and robust multistability. The simulations further suggest that the kinetics of differentiation to a mature cell state can depend on the starting progenitor state as well as on the route of commitment that is chosen. Lastly, our model shows good agreement with lineage-specific receptor expression kinetics from microarray experiments and provides a computational framework that can integrate both classical and alternative commitment paths in hematopoiesis that have been observed experimentally.

## Author Summary

Complex biomolecular interaction pathways in signaling networks can lead to non-intuitive behaviors that can prove critical for the regulation and robustness of biological processes. In this work, we present a signaling topology that can generate dynamic responses that are particularly pertinent to cell commitment in hematopoiesis. Our minimal model explores fundamental questions of instructive signaling that have persisted in cell-fate decisions. We show that even when lineage commitment decisions are inherently noisy, external cytokine signals, amplified by receptor upregulation, can bias the lineage choices of a progenitor cell. The multipotent progenitor, based on its differentiation potential, can exhibit several layers of memory to provide stability to both intermediate and mature states and can potentially bypass canonical intermediate states in generating mature cell types. Thus, our model provides a computational framework that can accommodate both classical and non-classical commitment paths in hematopoiesis.

## Introduction

Multipotent stem cells have the ability to both self-renew and differentiate, thus sustaining the stem cell pool and giving rise to mature, specialized cells, respectively. The hematopoietic stem cell (HSC), located in the adult bone marrow, is well characterized and has served as a popular model system for understanding self-renewal, lineage commitment, and differentiation [1]. HSCs are responsible for producing the entire repertoire of blood cells through the process of hematopoiesis. During hematopoiesis, HSCs lose the capacity to self-renew and differentiate into common myeloid progenitors (CMP) and common lymphoid progenitors (CLP) [2],[3]. Multipotent progenitors undergo further lineage-restricted differentiation to give rise to mature cells via bipotent progenitors. In addition to this classical commitment paradigm in hematopoiesis, alternative pathways are emerging. For example, it has also been observed that HSCs and multipotent progenitors can bypass canonical intermediate states during commitment [2],[4],[5]. The exact molecular events that direct lineage commitment at the stem cell stage or at the multipotent progenitor level remain elusive, but it is well appreciated that lineage-specific transcription factors and cytokine receptors play critical roles.

Lineage-specific transcription factors have been identified as master regulators of commitment and differentiation. They drive the expression of pertinent lineage-specific genes, thereby initiating the phenotypic change in the progenitor cell down a specific differentiation path [6],[7]. Developmental potency of a multipotent progenitor is reflected by the co-expression of multiple lineage-specific transcription factors at low levels, a phenomenon known as transcriptional priming [8]. This promiscuous gene expression pattern in the progenitor cell necessitates that, during cell differentiation, a specific transcription factor is upregulated, chiefly by positive autoregulation [9],[10], and other lineage transcription factors are downregulated, primarily through cross-antagonism [11]–[13].

In addition to lineage-specific transcription factors, cell differentiation is also believed to be tightly regulated by cytokines. Cytokines signal via their cognate receptors whose cytoplasmic domains activate various pathways involved in survival, proliferation, and differentiation [14]–[16]. It has been extensively debated whether cell fate during differentiation is a stochastic or an instructive process. The stochastic theory claims that the differential expression of lineage-specific transcription factors due to intrinsic noise in progenitor cells dictates the commitment decision [17]–[19], whereas the instructive theory argues that the absolute dependence on lineage-specific cytokine receptor signals during differentiation shows that cell-fate decisions are regulated by extrinsic growth factor cues [14],[15],[20],[21]. An underlying question evoked by both of these theories is whether cytokines provide instructive cues or select lineage-committed progenitors by providing permissive survival and proliferation signals. The instructive model does not account for the occurrence of certain mature cell types even when their lineage-specific receptors are knocked out [16],[18]. The predetermined distribution of the heterogeneous progenitor population into mature cells, as suggested by the stochastic model fails to explain how specific cell types can be enriched during stress or how homeostasis is restored after infections or therapy [15]. A recent landmark study utilizing bioimaging techniques at the single-cell level suggests that there is validity to both of these theories [20]. These authors showed that lineage-specific cytokines can strongly instruct lineage choice, although differentiation was still possible in the absence of lineage-specific cytokines.

A more comprehensive understanding of lineage commitment may emerge by analyzing the biochemical associations that coordinate cell-extrinsic and cell-intrinsic events. The promiscuous gene expression pattern during differentiation is observed not only in lineage-specific transcription factors, but also in lineage-specific receptors. A critical commitment signal during differentiation is the upregulation of the transcription factor, which aids in expressing the lineage-specific genes; however, the need to upregulate the lineage-specific receptor, an event also integral to commitment, is still unclear. This is particularly confounding since the low number of lineage-specific receptors present in a progenitor cell is sufficient for providing permissive survival cues. During lineage commitment, the expression of the cytokine receptor mirrors the expression of the transcription factor, often due to the presence of transcription factor binding domains in the promoter region of the receptor gene [22]–[25]. The advantage of regulating the lineage-specific receptor expression through the lineage-specific transcription factor is not apparent. Recent biochemical evidence also suggests that cytokines can provide signals to functionally activate lineage-specific transcription factors through post-translational modifications [26] and can also regulate the expression of transcription factors during cell differentiation [27].

Cell differentiation is believed to be an all-or-none “switch-like” event rather than a gradual transition of a precursor cell to a stable, mature cell. Mathematical modeling and analysis have been successfully used to provide insights into the biological networks that give rise to such switch-like behaviors [28]. Typically, the networks involved in lineage specification seem to engender cellular memory through nonintuitive behaviors, such as bistable response profiles. The components that generate bistability, the toggling of the system between two stable steady states, include nonlinear feedback loops [29],[30], external noise [31], and multi-site covalent modifications [32]. Previous lineage commitment models have suggested that transcriptionally primed multipotent progenitors are capable of exhibiting bistability purely via cell intrinsic events of autoregulation and cross-antagonism [8],[33],[34], but these models have assumed the existence of cooperative positive feedback loops to achieve bistability and do not consider the role of extracellular cues.

While cooperativity is a widely recognized biological mechanism that may play an important role in lineage commitment, alternative mechanisms can generate similar switch-like behavior in networks where cooperativity has not been observed. For example, we have previously shown that cytokine-regulated, positive feedback of receptor can generate robust bistability to stimulus without cooperativity in a deterministic model for unilineage commitment [35]. Furthermore, even in networks with cooperativity, receptor-mediated feedback may provide additional robustness to the system behavior and, perhaps more importantly, offer an external mode of regulation of cell-fates.

Here, we present a minimal model that integrates the bidirectional regulation between lineage-specific cytokines and transcription factors with previously explored autofeedback loops and cross-antagonism to understand the interplay between cell-extrinsic and cell-intrinsic factors in fate decisions of hematopoietic progenitors. Our model shows that the strength of cross-antagonism can be a critical determinant in achieving multistability. The analyzed network exhibits a “bilayer” of memory with respect to external stimuli to provide robustness to both the bipotent and committed states. The model suggests that noise in the network can enable stochastic switching between the stable states; however, the distribution of the uncommitted population among the various states during differentiation can still be strongly biased by external cues (as has now been experimentally observed [20]). Furthermore, this modeling framework captures both classical and alternative modes of lineage commitment seen in hematopoiesis. Although discrete cell fates are likely to represent high-dimensional attractors [34],[36], our minimal model may provide an initial step towards understanding how extrinsic factors integrate with intrinsic factors and may elucidate new mechanisms that underlie cell-fate decisions.

## Results

### Model formulation

Different cell states in our model are identified by the relative expression levels of lineage-specific receptors and transcription factors. An uncommitted (or ‘off-state’) cell, such as a common myeloid progenitor (CMP), is one that expresses lineage-specific receptors and transcription factors for multiple lineages at low levels. It is primed to differentiate into several lineages, but not yet committed to any specific lineage. A bipotent (or ‘intermediate-state’) cell, such as a megakaryocyte-erythrocyte progenitor (MEP), is one that is restricted to exactly two lineages, but not yet committed to either of them. Lineage-specific receptors and transcription factors for the two lineages are expressed at intermediate levels. A committed (or ‘on-state’) cell, such as a proerythroblast, is one that expresses the receptor and transcription factor of a single lineage at a high level and will eventually terminally differentiate into the corresponding mature cell.

The topology of our minimal model for multilineage commitment was informed by various experimental studies on lineage-specific receptors and transcription factors. The cytokines Epo, Tpo, GCSF, and MCSF have been shown to offer instructive cues to uncommitted and bipotent cells to differentiate into committed cells, which then terminally differentiate into erythrocytes, megakaryocytes, neutrophils, and macrophages, respectively [3],[15],[20],[37]. Lineage-specific transcription factors GATA-1, PU.1, T-bet, and GATA-3 orchestrate the differentiation program of erythrocytes, neutrophils, Th1, and Th2 cells, respectively, by regulating the expression of their lineage-specific genes [6],[38]. Transcription factors GATA-1 and PU.1 have been shown to autoregulate their gene expression by binding to the promoter region of their own genes [9],[10]. Erythrocytic transcription factor GATA-1 has been shown to transactivate the Epo receptor (EPOR) gene and the neutrophilic transcription factor PU.1 has been observed to regulate the expression of the GCSF receptor (GCSFR) [22],[25]. A transcription factor can prevent another transcription factor from binding to DNA either by competitively binding to response elements (as in the case of GATA-1 and GATA-2 [12]) or by binding to the DNA-binding domain of the transcription factor itself (for example, GATA-1 and PU.1 [13]).

**Figure 1:**
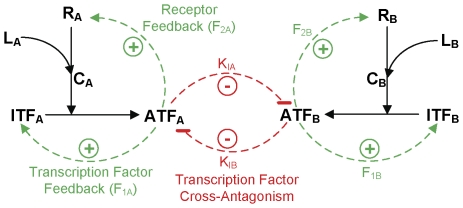
A minimal model of multilineage commitment. A multipotent progenitor expresses lineage-specific receptors (R_A_ and R_B_) and inactive transcription factors (ITF_A_ and ITF_B_) at low levels with the potential to differentiate into lineage A or B. Addition of ligand (L_A_, L_B_) leads to complex formation (C_A_, C_B_), which activates the corresponding lineage-specific transcription factor. Active TF (ATF_A_, ATF_B_) binds to the response elements present upstream of the transcription factor and receptor genes and induces two positive feedback loops (dashed green arrows). To account for cross-antagonism between the lineages, the active transcription factors are modeled to competitively inhibit the activation of the positive feedback loops in the other lineage (dashed red lines). F_1A_ and F_2A_ denote the respective strengths of the transcription factor and receptor feedback loops for lineage A; similarly, F_1B_ and F_2B_ represent the corresponding feedback strengths for lineage B. Inhibitor dissociation constants K_IA_ and K_IB_ denote the inhibitory effect of A on B and B on A, respectively.

The topology shown in Figure 1 represents a generalized minimal network of these observed connections between the cytokine and lineage-specific transcription factor during lineage commitment. The model assumes that the fate decision of an uncommitted cell to either lineage A or lineage B is determined solely by the concentrations of the active forms of the respective lineage-specific transcription factors, ATF_A_ and ATF_B_. The components that drive the formation of each ATF are the inactive transcription factor (ITF), which serves as the substrate, and the ligand (L)-receptor (R) complex (C), which serves as the enzyme. The strong upregulation of ATF during lineage commitment is achieved through two positive feedback loops that upregulate ITF and R, respectively. Transcription factor feedback is a cell-intrinsic autofeedback loop and receptor feedback is an externally (ligand) regulated positive feedback loop. F_1A_ and F_2A_ (expressed in molecules/min) denote the strengths of the transcription factor and receptor feedback loop for lineage A, respectively; F_1B_ and F_2B_ represent the corresponding feedback strengths for lineage B. During commitment, a lineage-specific transcription factor gets upregulated and other lineage transcription factors get downregulated due to cross-antagonism [11]–[13]. The mechanism of cross-antagonism between the transcription factors is modeled to be competitive inhibition in binding to response elements present upstream of the transcription factor and receptor genes, thereby affecting the strengths of the two positive feedback loops. While cell fates are likely to represent high dimensional attractors [34],[36] and this higher level of complexity is not considered here, our minimal model framework may be useful in elucidating the interplay among extrinsic and intrinsic factors in lineage commitment and differentiation. The deterministic (ordinary differential equations) and the stochastic (probability functions) versions of the model along with the kinetic parameters and initial conditions are provided in Supplementary Tables S1, S2, S3.

### Double positive feedback loops, coupled with moderate transcriptional cross-antagonism, can lead to multistability

**Figure 2:**
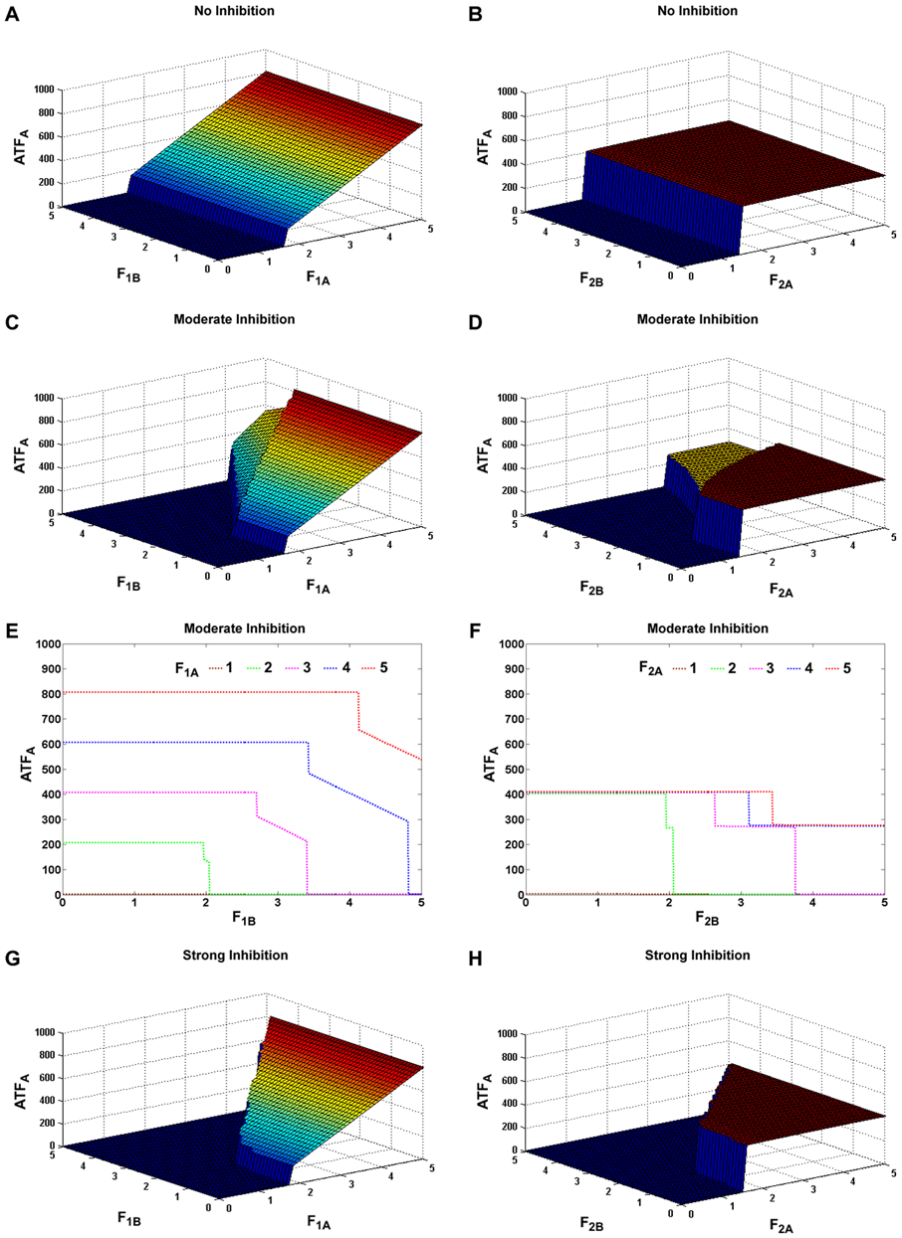
Effect of the positive feedback loops on the on-state ATF_A_ levels. A. Strengths of the autofeedback loops (F_1A_ and F_1B_) are varied for both lineages and the steady-state values of ATF_A_ are plotted for the no inhibition condition (K_IA_ = K_IB_ = ∞), keeping the strengths of the receptor feedback (F_2_) constant. B. Strengths of the receptor feedback loops (F_2A_ and F_2B_) are varied and the values of ATF_A_ are plotted for the no inhibition condition, keeping the strengths of the autofeedback (F_1_) constant. C. Same as part A except with moderate inhibition (K_IA_ = K_IB_ = 400 molecules). D. Same as part B except with moderate inhibition. E. Cross-sectional plot from C for various values of F_1A_. F. Cross-sectional plot from D for various values of F_2A_. G. Same as part A except with strong inhibition (K_IA_ = K_IB_ = 50 molecules). H. Same as part B except with strong inhibition. No inhibition and strong inhibition give rise to only on or off populations, whereas moderate inhibition can generate a third intermediate population.

To explore the role of the two positive feedback loops in lineage commitment, we first considered the case with no competitive inhibition between the transcription factors. The inhibitor dissociation constants K_IA_ (inhibitory effect of A on B) and K_IB_ (inhibitory effect of B on A) are kept infinite. Figure 2A shows the steady-state values of ATF_A_ as the strength of two autofeedback loops, F_1A_ and F_1B_, are changed. The strengths of the receptor-mediated feedback loops and the ligand levels are kept constant (F_2A_ = F_2B_ = 3 molecules/min, L_A_ = L_B_ = 100 molecules). We can see that the system rests in the uncommitted state when F_1A_ = 0 for the chosen F_2_ and L values. As we increase F_1A_, the system switches to the on-state (committed state) for lineage A. Since F_1_ constitutes the strength of the autofeedback loop in A, increasing F_1A_ over the threshold value will increase the set point of ATF_A_ in the on-state, provided F_2A_ is not limiting [35]. To consider the effect of receptor-mediated feedback on the steady-state values of ATF_A_, the strength of the autofeedback loops and ligand are kept constant (F_1A_ = F_1B_ = 3 molecules/min, L_A_ = L_B_ = 100 molecules). Similar to F_1A_, there seems to be a critical value for F_2A_ at which the system switches to the on-state (Figure 2B). As F_2_ controls the activation loop, increasing F_2A_ beyond the critical level will not change the on-state set point value of ATF_A_, provided F_2A_ is not limiting [35]. As expected, F_1B_ and F_2B_ have no effect on ATF_A_ since we have assumed no crosstalk between the two pathways.

The above analysis was repeated with moderate inhibition (K_IA_ = K_IB_ = 400 molecules). Similar to the no inhibition case, there appear to be critical values for F_1A_ and F_2A_ at which the system switches to the on-state (Figures 2C and 2D). However, increasing F_1B_ and F_2B_ increases the switching values of F_1A_ and F_2A_, due to the negative feedback from ATF_B_ on ATF_A_. It is interesting to note that for high values of F_1B_ and F_2B_, the system reaches a stable, intermediate state at which the concentration of ATF_A_ is higher than that in the uncommitted state, but less than that in the committed state (by symmetry, the same effect is observed for ATF_B_; see Supplementary Figure S1). As in the committed state, the set point in this intermediate state increases with F_1_, but not with F_2_. To better visualize the intermediate state, cross-sections of F_1B_ and F_2B_ from Figures 2C and 2D for various values of F_1A_ and F_2A_ are given in Figures 2E and 2F, respectively. For strong inhibition (K_IA_ = K_IB_ = 50 molecules), the system achieves commitment to lineage A for F_1A_ and F_2A_ values above the threshold levels. When F_1B_ and F_2B_ are increased over the critical value, the system requires concomitantly larger increases in F_1A_ and F_2A_ values to switch from the uncommitted state compared to the moderate inhibition condition (Figures 2G and 2H). Also, strong mutual inhibition between the transcription factors destroys the stable intermediate state, so the cells can rest only in the uncommitted or committed state. Since the model is symmetric with respect to lineages A and B, the steady-state responses of ATF_B_ with respect to changes in F_1_ and F_2_ are analogous to the results shown for ATF_A_ (see Supplementary Figure S1). It should be noted that the system is capable of achieving multistability for a given F_1_ and F_2_ ([35] and results not shown); however, only the stable solution attained without the memory of strong feedback is plotted in Figure 2 (i.e., the simulations were always started from the off-state).

### “Bilayer” memory in a tristable system

**Figure 3:**
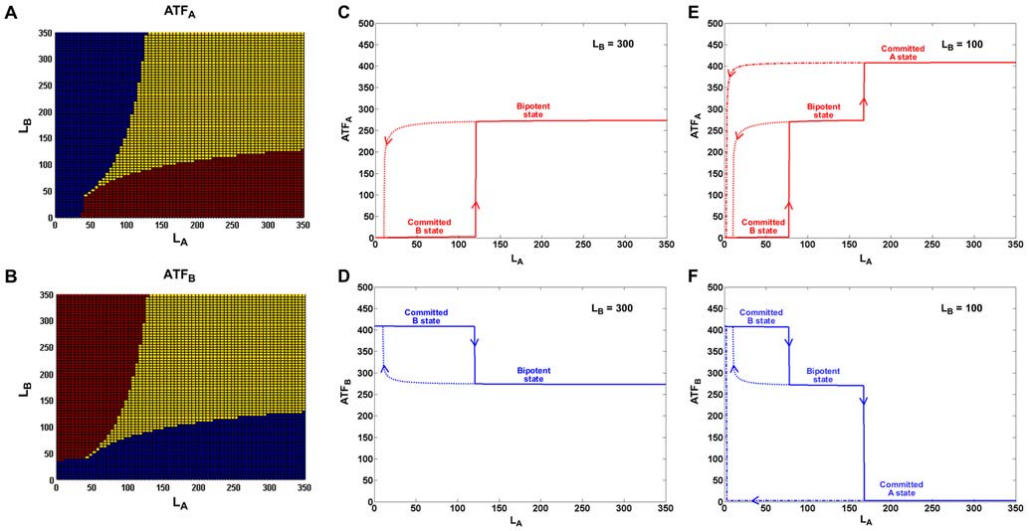
Effect of ligand on the on-state ATF levels. A. Phase plot showing the steady-state ATF_A_ levels (blue – low, yellow – medium, red – high) when L_A_ and L_B_ values are varied. B. Phase plot showing the steady-state ATF_B_ levels when L_A_ and L_B_ values are varied. Low L_A_ and low L_B_ do not commit the uncommitted cell to either lineage (overlapping blue region in panels A and B). Low L_A_ and high L_B_ values commit the cell to lineage B (blue region in panel A and red region in panel B). High L_A_ and low L_B_ values commit the cell to lineage A (red region in panel A and blue region in panel B). High L_A_ and high L_B_ commit the cell to the bipotent state (overlapping yellow region in panels A and B). Steady-state response plots: C. Increasing L_A_ from 0, with L_B_ constant at 300, abruptly switches the cell from the committed B state to the bipotent state (increase in ATF_A_ to intermediate level) after reaching a threshold concentration (solid red line). After achieving the bipotent state, decreasing L_A_ to sub-threshold values does not immediately switch the cell state, suggesting significant memory in the system (dotted red line). D. Increasing L_A_ from 0, with L_B_ constant at 300, decommits the cell to the bipotent state (decrease in ATF_B_ to intermediate level) after reaching the threshold concentration (solid blue line). After achieving the bipotent state, decreasing L_A_ to sub-threshold values does not immediately switch the cell state, again suggesting significant memory (dotted blue line). E. Increasing L_A_ from 0, with L_B_ constant at 100, abruptly switches the committed B cell to the bipotent state (increase in ATF_A_ to intermediate level) and then again to the committed A state (increase in ATF_A_ to high level) after reaching the corresponding threshold concentrations (solid red line). After achieving the bipotent state or the committed state, decreasing L_A_ to sub-threshold values does not immediately switch the cell response, suggesting significant memory in both states (dotted and dot-dash red line). F. Increasing L_A_ from 0, with L_B_ constant at 100, decommits the cell to the bipotent state (decrease in ATF_B_ to intermediate level) and then again to the committed lineage A state (decrease in ATF_B_ to low level) after reaching the corresponding threshold concentrations (solid blue line). After achieving the bipotent state or the committed lineage A state, decreasing L_A_ to sub-threshold values does not immediately switch the cell response, suggesting significant memory in both states (dotted and dot-dash blue line). Plots C and D show bistable expression of ATF_A_ and ATF_B_; plots E and F exhibit both bistable and tristable expression of the transcription factors.

External regulation provides a practical way to control the dynamics of the network without the need to alter the internal control elements of the system. We analyzed how cell commitment might be influenced in the presence of conflicting ligands with the strength of the positive feedback loops held constant (F_1_ = F_2_ = 3 molecules/min) for the moderate inhibition case. As seen from the phase plots in Figure 3, increasing L_A_ when L_B_ is low commits the uncommitted cell to lineage A (red region in Figure 3A), increasing L_B_ for low L_A_ commits the cell to lineage B (red region in Figure 3B), and for high values of both L_A_ and L_B_ the system rests at a third, bipotent state that is primed but not committed to either of the lineages (overlapping yellow regions in Figures 3A and 3B). For low L_A_ and L_B_ (both less than ∼40 molecules), the system remains in the uncommitted state (overlapping blue regions in Figures 3A and 3B).

To explore the robustness of the bipotent and committed states, we tested the system for memory to external stimulus. From the phase plots, we chose L_B_ = 300 to analyze the robustness of the bipotent state. The steady-state response plots of ATF_A_ and ATF_B_ for L_B_ = 300 are given in Figures 3C and 3D. In Figure 3C, increasing L_A_ switches the system from the committed B state to the bipotent state (solid red line). After reaching the bipotent state, the ligand concentration can be decreased far below the initial switching concentration while still maintaining the system in the bipotent state (dotted red line). However, complete removal of L_A_ switches the system back to the committed B state. For the ligand concentrations spanned by the dotted red line, the system is bistable. Considering the steady-state response of ATF_B_ in the same simulation, we see that for low L_A_ values, the system is already committed to lineage B (Figure 3D). However, increasing L_A_ can decommit the cell to a bipotent state (solid blue line). Decreasing L_A_ after reaching the bipotent state maintains the cell in that state for values of L_A_ much lower than the decommitment concentration (dotted blue line). So, the system is also bistable for ATF_B_ expression (inversely correlated to ATF_A_ expression) and can exist either in the committed state for lineage B or in the bipotent state based on the memory of L_A_.

To analyze the switching of the system across three states, we chose L_B_ = 100 based again upon the phase plots in Figures 3A and 3B. In Figure 3E, a modest increase in L_A_ switches the system to the bipotent state and a further increase in L_A_, switches the system to the committed A state (solid red line). If the ligand concentration is lowered after the system reaches either the bipotent state or the committed state, the system remains in the current state (dotted and dot-dash red lines). This hysteresis is greater for the committed state than for the bipotent state, suggesting that the committed state is more robust to changes in the ligand concentration. For L_B_ = 100 and for 10<L_A_<75, the system exhibits tristability (i.e., it can exist in committed state A, committed state B, or the bipotent state). The steady-state response plot of ATF_B_ for L_B_ = 100 (Figure 3F) shows that a committed B cell decommits to the bipotent state and then further to lineage A with an increase in L_A_ (solid blue line). As in Figure 3E, the bipotent and lineage A states are robust with respect to decreases in L_A_ (dotted and dot-dash blue lines) and the system exhibits tristability for the same concentration range of L_A_ as in Figure 3E. It should also be noted that the ligand-dependent multistability seen for a lineage-specific transcription factor is the same for the corresponding lineage-specific receptor, thus simultaneously generating memory in cell-extrinsic and cell-intrinsic signals [35].

### Extrinsic cues can regulate stochastic switching

**Figure 4:**
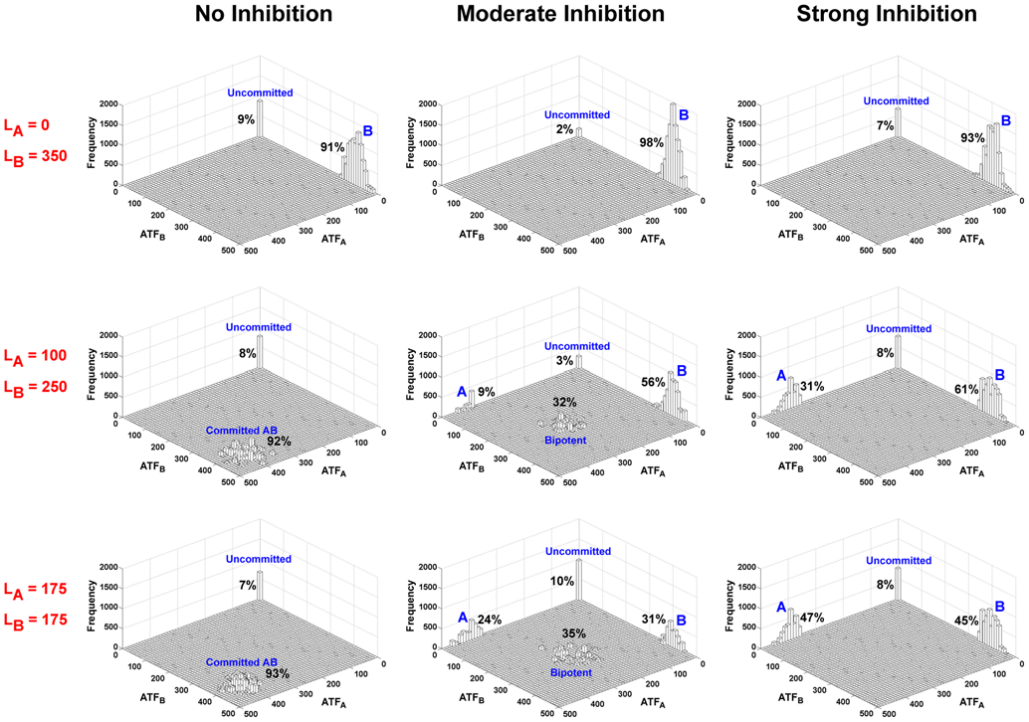
External regulation of stochastic transitions. Three different L_A_|L_B_ combinations (0|350, 100|250, and 175|175) were run using the stochastic version of the model with no, moderate, or strong inhibition conditions and the system was allowed to reach steady state. ATF_A_ and ATF_B_ values from 10,000 runs for each condition are plotted here as three-dimensional histograms. With strong inhibition, the system cannot achieve the intermediate, bipotent state that is seen with moderate inhibition. When induced with only one ligand (e.g., 0|350), the initial population, for all inhibition conditions, commits predominantly to the lineage corresponding to that ligand. When the uncommitted state is stimulated with equal values of ligand (175|175), the no inhibition condition primarily results in a state that corresponds to high activation of both transcription factors (unlikely to be a biologically relevant state for cell-commitment decisions); the strong and the moderate inhibition conditions result in significant population of all of the available states except the uncommitted state. When one ligand value is higher (e.g., 100|250), in the presence of inhibition, the majority of the cells committed to the lineage corresponding to the higher ligand concentration. The number next to each individual population denotes the percentage of the total population when treated with the given combination of L_A_ and L_B_.

We developed a stochastic version of the ordinary differential equation (ODE)-based deterministic model to analyze how noise in the network might affect the fate decision of an uncommitted cell (i.e., one that initially contains no ATF_A_ or ATF_B_) and how external signals might regulate these stochastic transitions. The stochastic model was initialized with several L_A_|L_B_ combinations (0|350; 100|250; 175|175; 250|100; 350|0) for the no inhibition, moderate inhibition, and strong inhibition conditions. In each of 10,000 simulations, the system was allowed to reach steady-state (see Supplementary Text S1) and steady-state ATF_A_ and ATF_B_ levels for the first three ligand combinations listed above are shown as 3D histograms in Figure 4 (since the model is symmetric, the 250|100 and 350|0 plots are virtual mirror images of the 100|250 and 0|350 plots, respectively, in Figure 4). Unlike the deterministic model, which only provided a population average of the four attainable steady states (uncommitted, bipotent, lineage A, lineage B) for any L_A_|L_B_, the stochastic simulations elucidated the relative populations of these multiple steady states for a given L_A_|L_B_. For the no inhibition condition, an uncommitted cell can reach any of four distinct stable states given the appropriate extracellular cues: uncommitted, A, B, and a committed AB state with high ATF_A_ and ATF_B_ values (though this last state is simply a consequence of having no inhibition and likely has little relevance in biological mechanisms specific to cell commitment decisions). When ATF_A_ and ATF_B_ can moderately inhibit each other, the uncommitted, A, B, and bipotent states can all be populated, even for a single L_A_|L_B_ combination (e.g., middle plot in Figure 4). However, when the transcription factors exhibit strong cross-antagonism, this bipotent state is no longer realizable and cells only commit fully to one lineage or the other or stayed uncommitted. The stochastic simulations with various combinations of conflicting ligand concentrations and for different levels of competitive inhibition show that all of the populations obtained from the deterministic model are stable and distinct even with the introduction of noise. For conditions in which only one ligand was present (e.g., 0|350), the cells committed only to the induced lineage for all levels of inhibition. A small fraction of the initial population remained uncommitted for all conditions for the chosen steady-state time point. When external cues of equal strength were provided (175|175), cells in the absence of inhibition primarily reached the committed AB state; with strong inhibition, they attained nearly equal levels of the committed A and B states; and with moderate inhibition, the cells were roughly evenly distributed across the bipotent, A, and B states. When high but unequal ligand levels were used (e.g., 100|250), cells in the no inhibition model commit almost exclusively to the AB state since the effects of L_A_ and L_B_ are entirely uncoupled. However for the strong and moderate inhibition conditions, the initial population committed predominantly to the lineage corresponding to the higher ligand value. This shows that, while the noise in the system is capable of distributing the initial population to all available steady states for any ligand concentration above a minimum threshold, a dominant external signal can still strongly bias the system to its specific lineage.

### Time trajectories during lineage commitment

**Figure 5:**
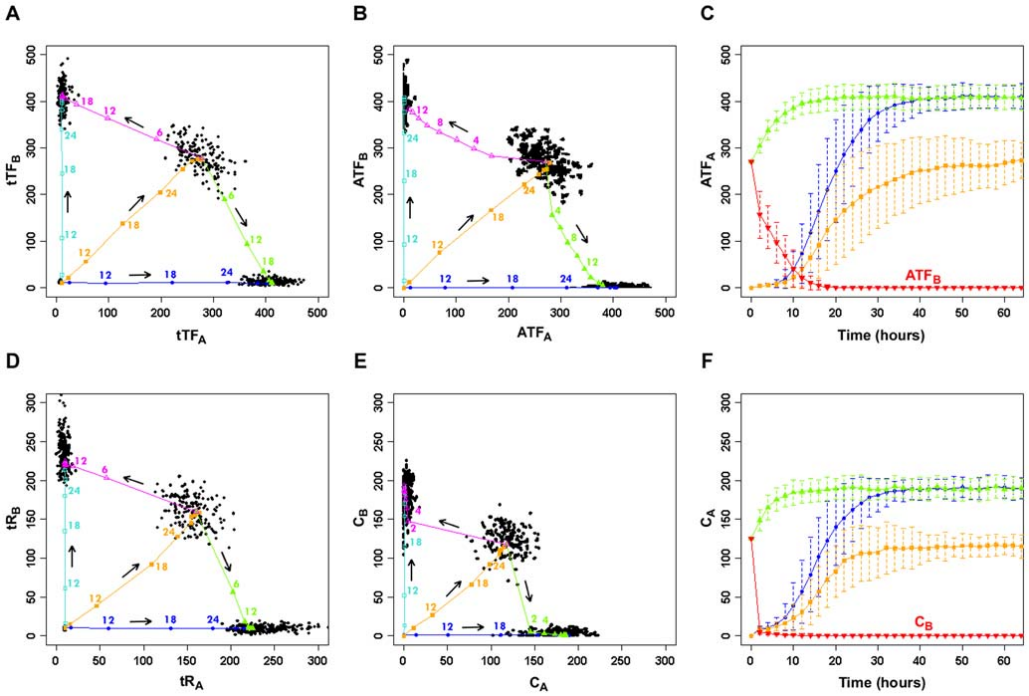
Time trajectories during lineage commitment. A. Phase plot of total transcription factor (ITF+ATF) for the four steady-state populations (uncommitted, A, B, and bipotent). B. Phase plot of active transcription factor (ATF). C. Time trajectories for ATF_A_ in panel B for the transition from the uncommitted cell to committed A state (blue line) and bipotent state (orange line) and from the bipotent state to committed A state (green line). The error bars represent the standard deviation of the mean. The red line shows the level of ATF_B_ as the bipotent cell transitions to the committed A state. D. Phase plot of total receptor (R+C). E. Phase plot of active complex (C). F. Time trajectories for C_A_ in panel E for the transition from the uncommitted cell to committed A state (blue line) and bipotent state (orange line) and from the bipotent state to committed A state (green line). The error bars represent the standard deviation of the mean. The red line shows the level of C_B_ as the bipotent cell transitions to the committed A state. In the phase plots, the arrows indicate the direction of commitment (averaged over 200 stochastic runs each): from the uncommitted state, the three possible commitment trajectories lead to pure lineage A, pure lineage B, and the bipotent state. In separate simulations starting with the bipotent state and with initial ligand concentrations sufficient to destabilize this state, the two possible commitment trajectories lead to pure lineage A and pure lineage B. Each trajectory has several nodes and the number at each node denotes the average time (in hours) it takes to reach the node from the initial state. Each black dot in A, B, D and E represents the endpoint (100,000 min) of an individual stochastic trajectory. The initial conditions for the trajectories are provided in the Supplementary Text S1.

From 100 individual stochastic trajectories, we calculated the average time for an uncommitted cell to reach lineage A, lineage B, or the bipotent state and, in separate simulations, the average time for a bipotent progenitor to reach lineage A or B. A phase plot of the total transcription factors (tTF = ITF+ATF) shows that it takes ∼36 hours for the uncommitted cell to reach lineage A, lineage B, or the bipotent state; however, when ligand concentrations that destabilize the bipotent progenitor are applied, it only takes ∼24 hours for the bipotent progenitor to reach either of the committed states (Figure 5A). This effect is even more pronounced when we look at the phase plots for ATF (Figure 5B); the time to reach the high level of active transcription factor(s) from the uncommitted cell is still ∼36 hours, however it takes much less time (∼14 hours) for the bipotent progenitor to reach lineage A or B. The kinetics of reaching new steady-state levels for total receptor (tR = R+C) and complex (Figures 5D and 5E) are faster than those for tTF and ATF, respectively, but the trend of reaching commitment faster from the bipotent state compared to the uncommitted state is similar to the transcription factor plots. Figures 5C and 5F respectively show the mean C_A_ and ATF_A_ values with respect to time (in hours) for transitions from the uncommitted state to lineage A (blue line), uncommitted state to bipotent state (orange line), and bipotent state to lineage A (green line). The error bars represent the standard deviation of the trajectories from the mean values. The red lines show the decreases in C_B_ and ATF_B_ as the bipotent cell follows the trajectory to commit to lineage A. A primed bipotent cell reaches either committed state faster than an uncommitted cell does, primarily due to the fact that accumulation of new transcription factor molecules (protein synthesis) is a much slower process than deactivation of existing active transcription factor molecules. Furthermore, cytokine signaling has been shown to accelerate differentiation [20], so the dynamics of activated receptors and transcription factors are likely to influence the kinetics of differentiation.

### Comparison to experiments

**Figure 6:**
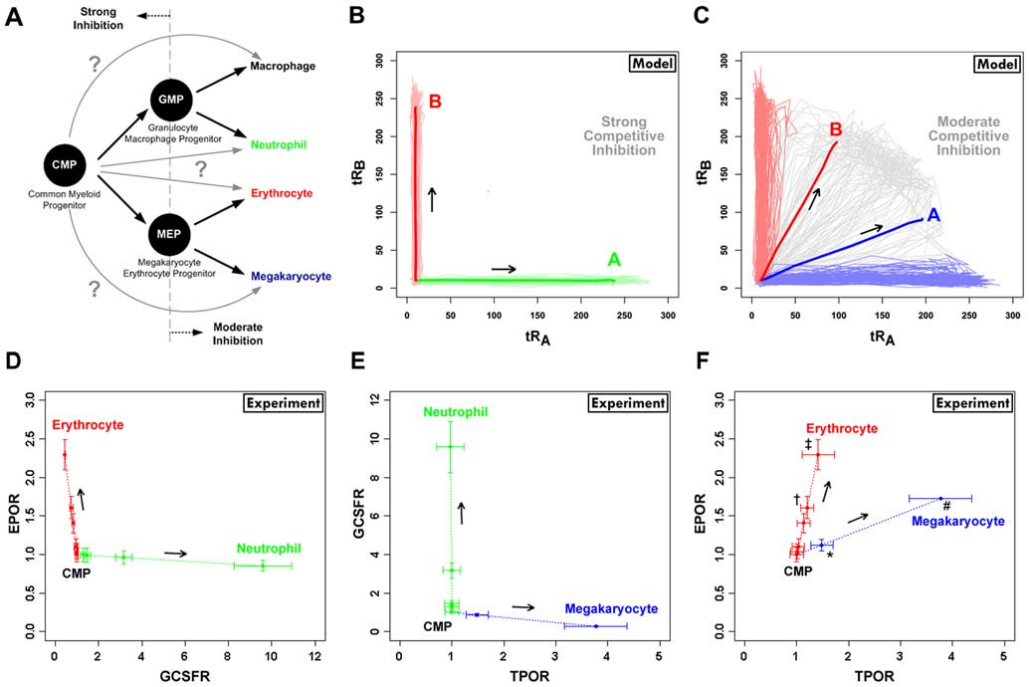
Comparison of multilineage commitment model to experimental data. A. The classical model of hematopoiesis is given here as a branching diagram showing the differentiation paths from the common myeloid progenitor (CMP) to four distinct myeloid lineages (megakaryocyte, erythrocyte, neutrophil, and macrophage) via bipotent progenitors (GMP – granulocyte/macrophage progenitor and MEP – megakaryocyte/erythrocyte progenitor). Potential non-canonical routes of commitment, bypassing the bipotent state, are shown as gray arrows. B. Stochastic simulations of total receptor levels under strong competitive inhibition. Light green and red lines indicate the individual trajectories from the uncommitted cell to lineages A and B, respectively. The dark red and green lines denote the averaged trajectories of all stochastic runs. C. Stochastic simulation for total receptor levels under moderate competitive inhibition condition. Light blue, light red, and gray lines indicate the individual trajectories from the uncommitted cell to A, B, and the bipotent state, respectively. The dark blue line denotes the average value of all stochastic runs that commit to either lineage A or the bipotent state; the dark red line denotes the average value of all stochastic runs that commit to either lineage B or the bipotent state. D. Trajectories from microarray data showing upregulation of EPOR and GCSFR during erythrocyte (red) and neutrophil (green) commitment from the CMP, respectively. E. Trajectories from microarray data showing upregulation of TPOR and GCSFR during megakaryocyte (blue) and neutrophil (green) commitment from the CMP, respectively. F. Trajectories from microarray data showing upregulation of EPOR and TPOR during erythrocyte (red) and megakaryocyte (blue) commitment from the CMP. The trajectories in D–F represent the average of the multipotent, bipotent, and mature cells for a single lineage (see Supplementary Table S4), thus enabling a direct comparison to the model simulations. The error bars in D–F show the standard error of the mean. The symbols in F denote the 3-day (†, *) and 7-day (‡, #) time points during erythrocyte and megakaryocyte differentiation from the CMP, respectively. Statistical analysis was performed to deduce positive correlation in receptor pair upregulation by comparing the overall slope of each trajectory (inverted to lie along the x-axis, if appropriate) at both the 3-day and 7-day time points to a value of zero (no correlation) by a one-sample, one-tailed t-test (p-values: † (0.027), * (0.009), ‡ (0.060), # (0.008)).

Figure 6A shows a widely accepted branching diagram for differentiation from the common myeloid progenitor (CMP). CMPs undergo lineage-restricted differentiation to form either granulocyte-macrophage progenitors (GMPs) or megakaryocyte-erythrocyte progenitors (MEPs). GMPs give rise to neutrophils or macrophages, whereas MEPs differentiate into megakaryocytes or erythrocytes. It has been recently demonstrated that alternative routes of differentiation are possible in hematopoiesis: HSCs and multipotent progenitors can bypass canonical intermediate states in reaching mature states [2],[4], suggesting that these lineage-restricting steps may be more complex than a series of simple binary decisions. We have shown analogous alternative trajectories in Figure 6A (gray arrows). In Figure 6B, the light green and light red lines represent 200 individual stochastic trajectories from the strong inhibition model that committed to lineage A and lineage B, respectively. The dark green and dark red lines show the average of these trajectories. As the strong inhibition model cannot generate a bipotent state, all of the trajectories are directed towards single-lineage populations (A or B). In Figure 6C, the light blue, gray, and light red lines denote 200 individual stochastic trajectories from the moderate inhibition model that committed to lineage A, the bipotent state, and lineage B, respectively. The dark blue line denotes the average of all trajectories committing to either lineage A or the bipotent state. The dark red line denotes the average of all trajectories committing to either lineage B or the bipotent state.

To qualitatively compare the receptor dynamics predicted by our model to those seen in experiments, we compared our simulations to lineage-specific receptor expression from microarray data (graciously provided from Bruno et al. [37] by Tariq Enver, University of Oxford); the data were collected at multiple time points during differentiation of multipotent myeloid progenitors (FDCP-mix, which are CMP-like progenitors [37]) across three lineages (neutrophil, erythrocyte, and megakaryocyte). The relative mRNA expression levels of the lineage-specific receptors – erythropoietin receptor (EPOR), granulocyte colony-stimulating factor receptor (GCSFR), and thrombopoietin receptor (TPOR) – were extracted from the processed microarray data. Since the phenotypic heterogeneity was also quantified at each time point in these microarray experiments [37], we were able to perform a simple deconvolution to estimate the contribution of each distinct cell type to the overall signal (see Supplementary Table S4). Therefore, the receptor expression trajectory for a given lineage in Figures 6D–F represents the average of only those multipotent, bipotent, and committed cells that lie along that specific lineage path (as is also the case for the average computational trajectories shown in bold lines in Figures 6B–C) and excludes those cells that belong to other commitment paths (for example, the TPOR trajectory includes blasts and megakaryocytes, but excludes erythroblasts, erythrocytes, and neutrophils which were also present in the *in vitro* cultures used for microarray analysis). The level of receptor was normalized to the basal levels in the CMP state. The error bars show the standard error of the mean from three independent experiments.

We constructed phase plots of EPOR and GCSFR showing the receptor trajectories (t = 0 to 7 days) as CMPs differentiate into either erythrocytes or neutrophils (Figure 6D). Induction of CMPs with EPO or GCSF drives cell commitment to the erythrocytic (red line) or the neutrophilic (green line) lineage, respectively [37]. During neutrophil commitment, GCSFR expression is significantly upregulated, but EPOR expression stays at or below basal levels; conversely, during erythrocyte commitment, EPOR expression is increased and GCSFR expression is unchanged or slightly reduced. Figure 6E shows the experimental phase plot of TPOR and GCSFR expression when CMPs are induced to differentiate into megakaryocyte or neutrophil lineages by stimulating with TPO (blue line) and GCSF (green line), respectively. As in Figure 6D, receptor expression corresponding to the induced lineage is upregulated and the receptor expression corresponding to the other lineage is unchanged or even slightly downregulated. Figure 6F shows the phase plot from the differentiation experiments to erythrocytic and megakaryocytic lineages. Induction of CMPs with Epo or Tpo drives CMPs to either the erythrocytic (red line) or the megakaryocytic (blue line) lineage. Interestingly, during erythrocyte and megakaryocyte commitment, EPOR and TPOR are co-upregulated; however, the observed increase was higher for the receptor corresponding to the specific lineage that was predominantly generated. Statistical analysis was performed to deduce positive receptor correlation for the receptor pairs in Figures 6D, 6E, and 6F by comparing the overall slope of each trajectory (inverted to lie along the x-axis, if appropriate) at both the 3-day and 7-day time points to a value of zero (no correlation) by a one-sample, one-tailed t-test. The correlation in receptor expression for EPOR-GCSFR and TPOR-GCSFR was either negative or not statistically significant. However, the EPOR-TPOR receptor pair showed a positive correlation with statistical significance. The symbols in Figure 6F denote the 3-day (†, *) and 7-day (‡, #) time points during erythrocyte and megakaryocyte differentiation from the CMP (p-values: † (0.027), * (0.009), ‡ (0.060), # (0.008)).

Comparing experimental results to the model simulations, we note that the trajectories in the erythrocyte-neutrophil (Figure 6D) and neutrophil-megakaryocyte (Figure 6E) plots compare well with the strong inhibition model (Figure 6B) and the trajectories from the erythrocyte-megakaryocyte plot (Figure 6F) show agreement with the moderate inhibition model (Figure 6C). This inference is validated by the widely accepted observation that the transcription factors for the erythrocytic and megakaryocytic lineages are strongly cross-antagonistic to the transcription factor for the neutrophil lineage [11]–[13]. Other than evolutionary constraints, the model suggests that the strength of the transcriptional cross-antagonism can dictate whether two distinct lineage-specific receptors (and the corresponding lineage-specific transcription factors) can be co-upregulated, which in turn can influence the nature of the instructive, possibly conflicting, cues that the cell receives. This paradigm may highlight different modes of receptor regulation, and corresponding transcriptional activity, in various stages and branches of hematopoiesis (e.g., Figure 6A).

## Discussion

Mathematical models of lineage commitment during hematopoiesis have generally analyzed cell-fate decisions from an intrinsic standpoint. Here, we show how extrinsic regulation can play a role in instructing lineage choice and, furthermore, how a cell might process and respond to conflicting extracellular cues. It has been extensively debated whether cytokines play an instructive or permissive role during lineage commitment. In this work, we show that cell-fate decisions can be stochastic but that external cues can strongly bias this stochasticity and instruct cells to specific lineages. A recent publication [20] definitively demonstrated an instructive role for cytokines in hematopoiesis. This strongly underscores our need to understand how extracellular cues, either in isolation or in combination, influence hematopoiesis. Our model also suggests a possible alternative mode of commitment, whereby an uncommitted multipotent progenitor may commit directly to a mature lineage without transitioning through a bipotent state. This potential plasticity has been seen experimentally in HSCs [4] and multipotent progenitors [2].

The initial cell state that is modeled here is a common multipotent progenitor that expresses multiple lineage-specific receptors and transcription factors at low levels and is capable of differentiating along several lineages. In particular, two lineages that may exhibit different levels of transcriptional cross-antagonism are analyzed. The lineage commitment decision is modeled to be driven by the accumulation of the functionally active form of the lineage-specific transcription factor. This event is driven through two positive feedback loops, a synthesis loop that produces the transcription factor and a regulatory loop that aids in the activation of the transcription factor. This two-step positive feedback mechanism provides a means to externally regulate the classical autofeedback loop and can be of general significance in cell-fate decision models. In our lineage commitment model, the regulatory loop targets the cell-surface receptor, but analogous topologies may be seen in systems where the regulation is achieved extracellularly (upregulating the ligand) or intracellularly (upregulating a rate-limiting enzyme in the signaling pathway). Also, it should be noted that even though we have considered the external stimuli to be cytokines, they may also be cell-cell interactions, cell-matrix interactions, mechanical cues, or other diffusible factors.

Through steady-state response plots, we have shown that the system exhibits ultrasensitivity to ligand and can achieve multistability in active transcription factor levels (Figure 3). Here, ultrasensitivity to ligand confers switch-like behavior in cell-fate specification. Multistability provides memory to both the intermediate (bipotent) and committed cell states, enabling the system to robustly sustain its current state even when external stimuli are reduced to sub-threshold levels. Although the system modeled here represents a reversible switch, irreversibility during differentiation can be achieved by epigenetic means such as chromatin remodeling.

In support of the stochastic theory of commitment, our model suggests that, irrespective of the strength of external factors, intrinsic noise in transcriptional networks can switch a significant percentage of cells to a committed state or the bipotent state; however, in support of the instructive theory, extrinsic cues can still strongly bias the majority of the uncommitted cell population to the final state induced by the higher ligand signal, as seen in Figure 4. This figure also highlights how the same network topology can generate both binary and ternary cell-fate decisions. For example, strong inhibition enables only a binary cell-fate choice; however, simply relaxing the strength of the inhibition to moderate levels enables three possible fates from the uncommitted state.

**Figure 7:**
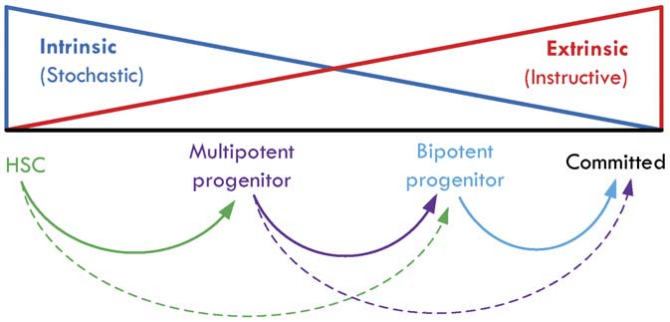
Proposed paradigm for hematopoiesis. Extrinsic (instructive) and intrinsic (stochastic) cues can both play roles in commitment of progenitor cells. In addition to classical pathways of commitment (solid arrows), bypass mechanisms have been reported for HSCs [4] (dashed green arrow) and our model suggests that this may be possible for multipotent progenitors as well (dashed purple arrow).

Our model suggests a new paradigm that integrates classical and alternative modes of lineage commitment and also accommodates both stochastic and instructive roles in hematopoiesis (Figure 7). It is generally appreciated that upstream commitment events are more stochastic in nature while downstream events are more instructive. Stochastic events in HSCs and multipotent progenitors can potentially lead to the generation of all mature cell types, explaining ‘normal’ hematopoiesis even when a lineage-specific receptor is knocked out [16],[18] (although other non-canonical extrinsic cues may also play compensatory roles). In parallel, instructive cytokine signaling in multipotent progenitors and bipotent progenitors, which can strongly bias and accelerate lineage commitment [20], may drive stress responses and restore homeostasis [15]. Furthermore, emerging alternative commitment paths suggest that decision-making in hematopoietic progenitors need not be purely binary. HSCs have been shown to bypass multipotent progenitors and directly produce bipotent MEPs [4] and common lymphoid progenitors appear to directly generate T cells, B cells, and NK cells [2]. The model presented in this work suggests a framework in which both binary and ternary decisions may be possible in multipotent CMPs. Such bypass mechanisms in commitment may also provide important redundancies that ensure mature cell production if a specific intermediate state becomes dysregulated.

Many of the predictions from our minimal, multipotent commitment model can be experimentally verified. Multipotent and bipotent progenitors can be identified and isolated with multi-color fluorescence-activated cell sorting, using specific cell-surface markers for the lineages of interest. Cytokine-induced time course experiments conducted on these bipotent cells can corroborate whether they reach mature states faster than the corresponding multipotent progenitors. Experiments with conflicting extracellular ligand cues can be useful in determining the strength of the instructive cues, the degree of transcriptional cross-antagonism between lineages, and the existence of a bipotent progenitor. For example, to analyze the differentiation paths of erythrocytes and neutrophils from a common progenitor, FDCP-mix cells can be induced with both Epo and GCSF and the trajectories of the expression of the lineage-specific transcription factors (GATA1, PU.1) and receptors (EPOR, GCSFR) can be determined by sensitive flow cytometry measurements. Groundbreaking new bioimaging techniques which enable observation of single cells over an extended period [20] should mitigate technical difficulties that have hampered such analyses and should help to further elucidate the roles of extrinsic and intrinsic regulation on cell commitment decisions.

## Methods

The minimal model shown in Figure 1 represents a regulatory network for lineage commitment of a multipotent progenitor to lineages A and B. The multipotent progenitor expresses basal levels of both lineage-specific transcription factors TF_A_ and TF_B_ (present in their inactive forms ITF_A_ and ITF_B_) and lineage-specific receptors R_A_ and R_B_ before the addition of ligand. Addition of L_A_ to the system leads to receptor-ligand complex C_A_ formation. Complex C_A_ activates signaling pathways that lead to the activation of ITF_A_ to form ATF_A_. Even though a mechanistic understanding of how this occurs via cytokine-mediated signaling has not fully emerged, we have modeled it to be rapidly regulated at the protein level (e.g., by post-translational modification). There may be other mechanisms involved (e.g., transcriptional and translational regulation) that are not considered here. The activated form of the transcription factor, ATF_A_, upregulates the transcription of its own gene through a positive autoregulatory feedback loop, enhancing production of ITF_A_. ATF_A_ also upregulates the expression of the lineage-specific receptor R_A_ forming a ligand-regulated positive feedback loop. The model also accounts for basal synthesis of R_A_ and ITF_A_, degradation of R_A_, C_A_, ITF_A_ and ATF_A_ and inactivation of ATF_A_ (not explicitly shown in Figure 1). For simplicity, we consider the network topology in the commitment of the two lineages to be symmetric: the reactions involved in the activation of ITF_B_ to ATF_B_ by ligand L_B_ and the formation of the two positive feedback loops are analogous to those described in lineage A. To account for the cross-antagonism between the transcription factors TF_A_ and TF_B_, ATF_A_ and ATF_B_ are modeled to downregulate the induced expression of [ITF_B_, R_B_] and [ITF_A_, R_A_] by competitively inhibiting the binding of ATF_B_ and ATF_A_ to the regulatory domains present upstream of their lineage-specific receptor and TF genes. This multilineage commitment network led to a deterministic model with eight ordinary differential equations (ODEs), shown in Supplementary Table S1. The initial conditions and the values of the rate constants are provided in Supplementary Table S2. A single-compartmental homogenous system is assumed and the pathways involved in TF activation and in the synthesis of TF and receptor are lumped as single-step reactions.

### Stochastic version of the deterministic model

The Gillespie stochastic algorithm was employed to simulate a stochastic version of the ODE model [39]. The stochastic reactions and their probability functions are given in Supplementary Table S3. Conversion of the deterministic model to its stochastic form was performed by using composite Michaelis-Menten type rate expressions in the propensity function instead of decomposing the minimal model into a series of elementary reactions; this was done to directly compare the dynamics of both the approaches [40],[41]. A detailed description of the stochastic simulations, including the parameter values, initial conditions, and the number of runs for Figures 4, 5, and 6, is provided in the Supplementary Text S1.

### Computational methods

The ODE-based deterministic model was solved using the numerical stiff solver ode15s in MATLAB (The Mathworks, Natick, MA). Time course, steady-state response and multistability plots were also created using MATLAB. The Gillespie algorithm for the stochastic model was programmed in C++. Histograms, phase plots and time trajectories of the stochastic simulations were created using the open-source statistical package R.

### Microarray analysis

Normalized microarray data from Bruno et al. [37] were generously provided by Tariq Enver (University of Oxford). The detailed experimental procedures for the microarray experiments and analyses are provided elsewhere [37]. EPOR, GCSFR and TPOR mRNA levels extracted from the data were further normalized to their basal levels present in the uninduced FDCP-mix. The inherent heterogeneity in the differentiating populations at each time point was overcome by weighting the contribution of each cell population to the average expression of the gene of interest. A detailed description and analysis of the weighting function used and the fitted parameters for the individual genes are provided in Supplementary Table S4.

## Supporting Information

Text S1 Supporting text
(0.07 MB PDF)

Figure S1 Effect of positive feedback loops on the steady-state level of ATF_B_ for different levels of transcriptional cross-antagonism
(0.10 MB PDF)

Table S1 Ordinary differential equations for the deterministic model
(0.06 MB PDF)

Table S2 Rate constants and initial conditions for the deterministic and stochastic models
(0.08 MB PDF)

Table S3 Probability functions and reactions for the stochastic model
(0.04 MB PDF)

Table S4 Parameter fitting of microarray data
(0.09 MB PDF)
