# Supplementary material for: Integrating Extrinsic and Intrinsic Cues into a Minimal Model of Lineage Commitment for Hematopoietic Progenitors
Source: PLoS Comput Biol. 2009 Sep 25;5(9):e1000518. doi: 10.1371/journal.pcbi.1000518 (PMC2736398; doi:10.1371/journal.pcbi.1000518)
Supplement: Text S1 — Supporting text (0.07 MB PDF) [file pcbi.1000518.s001.pdf]

SUPPLEMENTARY TEXT FOR:

Integrating extrinsic and intrinsic cues into a minimal model of lineage commitment for hematopoietic progenitors

Santhosh Palani[*] and Casim A. Sarkar[*†‡]

[*]Department of Bioengineering, [†]Department of Chemical & Biomolecular Engineering, University of Pennsylvania, Philadelphia, Pennsylvania 19104-6321 USA

Running Title: Model of multilineage commitment

[‡]**Author to whom correspondence should be addressed**
240 Skirkanich Hall, 210 S. 33rd Street,
Department of Bioengineering, University of Pennsylvania,
Philadelphia, PA 19104-6321
Tel: 215-573-4072
Fax: 215-573-2071
E-mail: casarkar@seas.upenn.edu

Stochastic model

The probability functions and reactions for the stochastic model are provided in Table S3. The model was run either from the uncommitted state or from the bipotent state to reach the committed state. The initial conditions for both starting states are also provided in Table S3.

To achieve values close to the true stable steady states, the simulations for both the deterministic and the stochastic models were run for 100,000 mins or ~70 days. As an example, for one representative set of parameter values, the deterministic model reached 99% of its 100,000-minute value at approximately 2500 mins. Thus, even for conditions that took significantly longer to reach steady state, our simulation time was more than sufficient. Also, since the time point that we picked is arbitrary, we performed 100 repetitions for each simulation condition to account for random fluctuations. Around this time point, the noise distribution had low variance and was relatively constant.

Small perturbations in the initial conditions for active species have no effect on the steady-state values or the distribution among the final states. Large perturbations also have no effect on the steady-state values, but can significantly alter the final-state distribution by priming the system with active receptors and/or transcription factors.

Parameters used to generate Figure 4

All simulations were started from the uncommitted state with $L_A|L_B$ values of 0|350, 100|250, 175|175, 250|100 and 350|0.

Initial conditions of the reactants:
$[R_A]_0 = 10$; $[R_B]_0 = 10$; $[C_A]_0 = 0$; $[C_B]_0 = 0$; $[ITF_A]_0 = 10$; $[ITF_B]_0 = 10$; $[ATF_A]_0 = 0$; $[ATF_B]_0 = 0$

For no inhibition condition: $K_{IA} = K_{IB} = \infty$
For moderate inhibition condition (in molecules): $K_{IA} = K_{IB} = 400$
For strong inhibition condition (in molecules): $K_{IA} = K_{IB} = 50$

All other parameter values are given in Table S2. For each condition, the simulations were run for 100,000 minutes and repeated 10,000 times.

Parameters used to generate Figure 5

All simulations were run using the moderate inhibition condition (in molecules): $K_{IA} = K_{IB} = 400$

Initial conditions of the reactants starting from the uncommitted state:
$[R_A]_0 = 10$; $[R_B]_0 = 10$; $[C_A]_0 = 0$; $[C_B]_0 = 0$; $[ITF_A]_0 = 10$; $[ITF_B]_0 = 10$; $[ATF_A]_0 = 0$; $[ATF_B]_0 = 0$
Initial conditions of the reactants starting from the bipotent state:
$[R_A]_0 = 30$; $[R_B]_0 = 30$; $[C_A]_0 = 125$; $[C_B]_0 = 125$; $[ITF_A]_0 = 6$; $[ITF_B]_0 = 6$; $[ATF_A]_0 = 273$; $[ATF_B]_0 = 273$

$L_A|L_B$ values for different trajectories:

Uncommitted to committed A – 250|5
Uncommitted to committed B – 5|250
Uncommitted to bipotent – 250|250
Bipotent to committed A – 250|5
Bipotent to committed B – 5|250

All other parameter values are given in Table S2. For each condition, the simulations were run for 100 hours and repeated 100 times.

Parameters used to generate Figures 6B and 6C

All simulations were started from the uncommitted state with $L_A|L_B$ values of 175|175.

Initial conditions of the reactants starting from the uncommitted state:
$[R_A]_0 = 10$; $[R_B]_0 = 10$; $[C_A]_0 = 0$; $[C_B]_0 = 0$; $[ITF_A]_0 = 10$; $[ITF_B]_0 = 10$; $[ATF_A]_0 = 0$; $[ATF_B]_0 = 0$

For moderate inhibition condition (in molecules): $K_{IA} = K_{IB} = 400$
For strong inhibition condition (in molecules): $K_{IA} = K_{IB} = 50$

All other parameter values are given in Table S2. For each condition, the simulations were run for 100,000 minutes and repeated 200 times.
