# Supplementary material for: Integrating Extrinsic and Intrinsic Cues into a Minimal Model of Lineage Commitment for Hematopoietic Progenitors
Source: PLoS Comput Biol. 2009 Sep 25;5(9):e1000518. doi: 10.1371/journal.pcbi.1000518 (PMC2736398; doi:10.1371/journal.pcbi.1000518)

Figure S1: Effect of positive feedback loops on the steady-state level of ATF_B for different levels of transcriptional cross-antagonism

Strengths of the autofeedback loops (F_{1A} and F_{1B}) are varied for both lineages and the steady-state values of ATF_B are plotted for no (A), moderate (C) and strong (E) inhibition, keeping the strength of receptor feedback (F_{2A} and F_{2B}) constant.

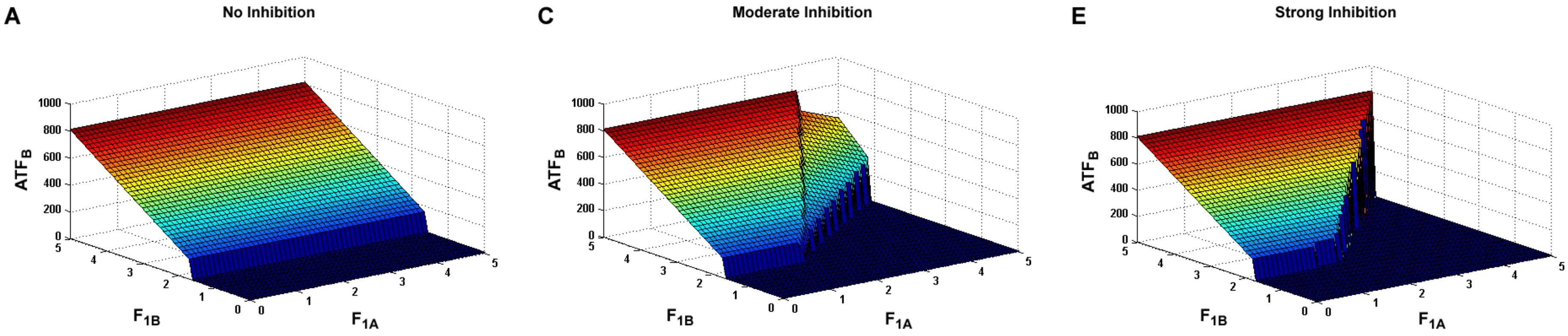

Strengths of the receptor feedback loops (F_{2A} and F_{2B}) are varied and the values of ATF_B are plotted for no (B), moderate (D) and strong (F) inhibition, keeping the strength of autofeedback (F_{1A} and F_{1B}) constant.

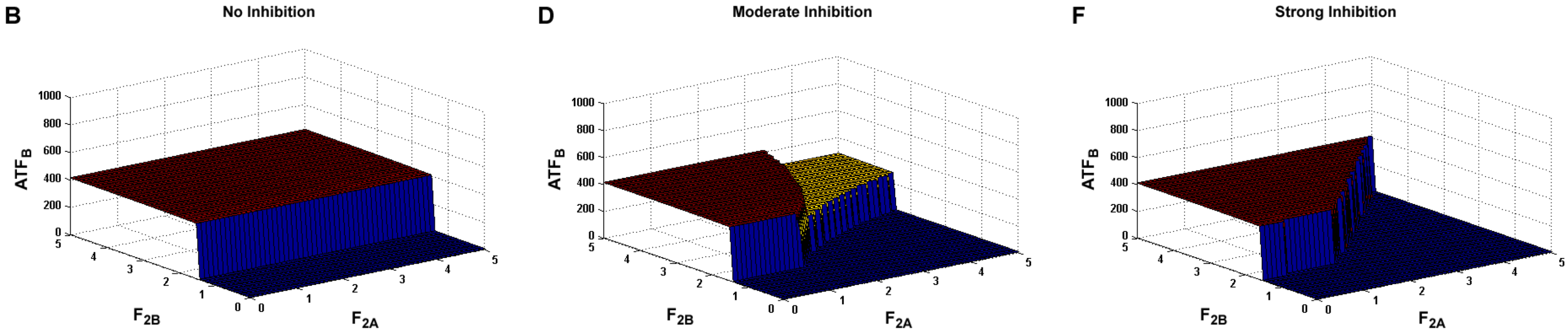

Supplement: Figure S1 — Effect of positive feedback loops on the steady-state level of ATFB for different levels of transcriptional cross-antagonism (0.10 MB PDF) [file pcbi.1000518.s002.pdf]
