# Supplementary material for: Integrating Extrinsic and Intrinsic Cues into a Minimal Model of Lineage Commitment for Hematopoietic Progenitors
Source: PLoS Comput Biol. 2009 Sep 25;5(9):e1000518. doi: 10.1371/journal.pcbi.1000518 (PMC2736398; doi:10.1371/journal.pcbi.1000518)
Supplement: Table S1 — Ordinary differential equations for the deterministic model (0.06 MB PDF) [file pcbi.1000518.s003.pdf]

Table S1: Differential equations for the deterministic model

$$\frac{d\left(R_A\right)}{dt} = B_A^R - kdegR_A * R_A - kon_A * L_A * R_A + koff_A * C_A + \frac{F_{2A} * ATF_A}{K_{DA} * \left[1 + \frac{ATF_B}{K_{IB}}\right] + ATF_A}$$

$$\frac{d\left(C_A\right)}{dt} = kon_A * L_A * R_A - koff_A * C_A - ke_A * C_A$$

$$\frac{d\left(ITF_A\right)}{dt} = B_A^{ITF} - kdegTF_A * ITF_A - \frac{k_{1A} * C_A * ITF_A}{K_{M1A} + ITF_A} + \frac{k_{2A} * P_A * ATF_A}{K_{M2A} + ATF_A} + \frac{F_{1A} * ATF_A}{K_{DA} * \left[1 + \frac{ATF_B}{K_{IB}}\right] + ATF_A}$$

$$\frac{d\left(ATF_A\right)}{dt} = \frac{k_{1A} * C_A * ITF_A}{K_{M1A} + ITF_A} - \frac{k_{2A} * P_A * ATF_A}{K_{M2A} + ATF_A} - kdegTF_A * ATF_A$$

$$\frac{d\left(R_B\right)}{dt} = B_B^R - kdegR_B * R_B - kon_B * L_B * R_A + koff_B * C_B + \frac{F_{2B} * ATF_B}{K_{DB} * \left[1 + \frac{ATF_A}{K_{IA}}\right] + ATF_B}$$

$$\frac{d\left(C_B\right)}{dt} = kon_B * L_B * R_B - koff_B * C_B - ke_B * C_B$$

$$\frac{d\left(ITF_B\right)}{dt} = B_B^{ITF} - kdegTF_B * ITF_B - \frac{k_{1B} * C_B * ITF_B}{K_{M1B} + ITF_B} + \frac{k_{2B} * P_B * ATF_B}{K_{M2B} + ATF_B} + \frac{F_{1B} * ATF_B}{K_{DB} * \left[1 + \frac{ATF_A}{K_{IA}}\right] + ATF_B}$$

$$\frac{d\left(ATF_B\right)}{dt} = \frac{k_{1B} * C_B * ITF_B}{K_{M1B} + ITF_B} - \frac{k_{2B} * P_B * ATF_B}{K_{M2B} + ATF_B} - kdegTF_B * ATF_B$$

L_A : Ligand for lineage A (constant)
R_A : Lineage-specific receptor for the A lineage
C_A : Ligand-receptor complex for the A lineage
ITF_A : Lineage-specific inactive transcription factor for the A lineage
ATF_A : Active transcription factor for the A lineage
P_A : Phosphatase deactivating ATF_A (constant)

L_B : Ligand for lineage B (constant)
R_B : Lineage-specific receptor for the B lineage
C_B : Ligand-receptor complex for the B lineage
ITF_B : Lineage-specific inactive transcription factor for the B lineage
ATF_B : Active transcription factor for the B lineage
P_B : Phosphatase deactivating ATF_B (constant)
