# Supplementary material for: Integrating Extrinsic and Intrinsic Cues into a Minimal Model of Lineage Commitment for Hematopoietic Progenitors
Source: PLoS Comput Biol. 2009 Sep 25;5(9):e1000518. doi: 10.1371/journal.pcbi.1000518 (PMC2736398; doi:10.1371/journal.pcbi.1000518)
Supplement: Table S2 — Rate constants and initial conditions for the deterministic and stochastic models (0.08 MB PDF) [file pcbi.1000518.s004.pdf]

Table S2: Rate constants and initial conditions for the deterministic and stochastic models

Rate constant	Value	Description
B_A^R	0.05 molecules/min	Basal synthesis rate of R_A
$kdegR_A$	0.005 min^{-1}	Degradation rate of R_A
kon_A	0.001 $molecules^{-1}min^{-1}$	Association rate constant for L_A and R_A binding
$koff_A$	0.05 min^{-1}	Dissociation rate constant for L_A and R_A binding
ke_A	0.01 min^{-1}	Endocytic rate constant for C_A
B_A^{ITF}	0.05 molecules/min	Basal synthesis rate of ITF_A
$kdegTF_A$	0.005 min^{-1}	Degradation rate of ITF_A and ATF_A
k_{1A}	60 min^{-1}	Rate of activation of ITF_A
K_{M1A}	100 molecules	Michaelis constant for activation of ITF_A
K_{2A}	30 min^{-1}	Rate of deactivation of ATF_A
K_{M2A}	30 molecules	Michaelis constant for deactivation of ATF_A
F_{1A}	3 molecules/min	Strength of transcription factor feedback for lineage A
F_{2A}	3 molecules/min	Strength of receptor feedback for lineage A
K_{DA}	200 molecules	Equilibrium dissociation constant for ATF_A binding to DNA
K_{IB}	400 molecules	Inhibitor dissociation constant (effect of B on A)
B_B^R	0.05 molecules/min	Basal synthesis rate of R_B
$kdegR_B$	0.005 min^{-1}	Degradation rate of R_B
kon_B	0.001 $molecules^{-1}min^{-1}$	Association rate constant for L_B and R_B binding
$koff_B$	0.05 min^{-1}	Dissociation rate constant for L_B and R_B binding
ke_B	0.01 min^{-1}	Endocytic rate constant for C_B
B_B^{ITF}	0.05 molecules/min	Basal synthesis rate of ITF_B
$kdegTF_B$	0.005 min^{-1}	Degradation rate of ITF_B and ATF_B
k_{1B}	60 min^{-1}	Rate of activation of ITF_B
K_{M1B}	100 molecules	Michaelis constant for activation of ITF_B
K_{2B}	30 min^{-1}	Rate of deactivation of ATF_B
K_{M2B}	30 molecules	Michaelis constant for deactivation of ATF_B
F_{1B}	3 molecules/min	Strength of transcription factor feedback for lineage B
F_{2B}	3 molecules/min	Strength of receptor feedback for lineage B
K_{DB}	200 molecules	Equilibrium dissociation constant for ATF_B binding to DNA
K_{IA}	400 molecules	Inhibitor dissociation constant (effect of A on B)

Phosphatases P_A and P_B are held constant at 15 molecules for all simulations.

Initial conditions when starting from the uncommitted or off-state (in molecules):

$[R_A]_0 = 10$; $[R_B]_0 = 10$; $[C_A]_0 = 0$; $[C_B]_0 = 0$; $[ITF_A]_0 = 10$; $[ITF_B]_0 = 10$; $[ATF_A]_0 = 0$; $[ATF_B]_0 = 0$

Initial conditions when starting from the bipotent or intermediate state (in molecules):

$[R_A]_0 = 30$; $[R_B]_0 = 30$; $[C_A]_0 = 125$; $[C_B]_0 = 125$; $[ITF_A]_0 = 6$; $[ITF_B]_0 = 6$; $[ATF_A]_0 = 273$; $[ATF_B]_0 = 273$
