# Supplementary material for: Integrating Extrinsic and Intrinsic Cues into a Minimal Model of Lineage Commitment for Hematopoietic Progenitors
Source: PLoS Comput Biol. 2009 Sep 25;5(9):e1000518. doi: 10.1371/journal.pcbi.1000518 (PMC2736398; doi:10.1371/journal.pcbi.1000518)
Supplement: Table S3 — Probability functions and reactions for the stochastic model (0.04 MB PDF) [file pcbi.1000518.s005.pdf]

Table S3 : Probability functions and reactions for the stochastic model

$\xrightarrow{P_1} R_A$	$P_1 = B_A^R$	$\xrightarrow{P_{13}} R_B$	$P_{13} = B_B^R$
$R_A \xrightarrow{P_2}$	$P_2 = kdegR_A * R_A$	$R_B \xrightarrow{P_{14}}$	$P_{14} = kdegR_B * R_B$
$R_A \xrightarrow{P_3} C_A$	$P_3 = kon_A * L_A * R_A$	$R_B \xrightarrow{P_{15}} C_B$	$P_{15} = kon_B * L_B * R_B$
$C_A \xrightarrow{P_4} R_A$	$P_4 = koff_A * C_A$	$C_B \xrightarrow{P_{16}} R_B$	$P_{16} = koff_B * C_B$
$C_A \xrightarrow{P_5}$	$P_5 = ke_A * C_A$	$C_B \xrightarrow{P_{17}}$	$P_{17} = ke_B * C_B$
$\xrightarrow{P_6} ITF_A$	$P_6 = B_A^{ITF}$	$\xrightarrow{P_{18}} ITF_B$	$P_{18} = B_B^{ITF}$
$ITF_A \xrightarrow{P_7}$	$P_7 = kdegTF_A * ITF_A$	$ITF_B \xrightarrow{P_{19}}$	$P_{19} = kdegTF_B * ITF_B$
$ITF_A \xrightarrow{P_8} ATF_A$	$P_8 = \frac{k_{1A} * C_A * ITF_A}{K_{M1A} + ITF_A}$	$ITF_B \xrightarrow{P_{20}} ATF_B$	$P_{20} = \frac{k_{1B} * C_B * ITF_B}{K_{M1B} + ITF_B}$
$ATF_A \xrightarrow{P_9} ITF_A$	$P_9 = \frac{k_{2A} * P_A * ATF_A}{K_{M2A} + ATF_A}$	$ATF_B \xrightarrow{P_{21}} ITF_B$	$P_{21} = \frac{k_{2B} * P_B * ATF_B}{K_{M2B} + ATF_B}$
$ATF_A \xrightarrow{P_{10}}$	$P_{10} = kdegTF_A * ATF_A$	$ATF_B \xrightarrow{P_{22}}$	$P_{22} = kdegTF_B * ATF_B$
$\xrightarrow{P_{11}} R_A$	$P_{11} = \frac{F_{2A} * ATF_A}{K_{DA} * \left[1 + \frac{ATF_B}{K_{IB}}\right] + ATF_A}$	$\xrightarrow{P_{23}} R_B$	$P_{23} = \frac{F_{2B} * ATF_B}{K_{DB} * \left[1 + \frac{ATF_A}{K_{IA}}\right] + ATF_B}$
$\xrightarrow{P_{12}} ITF_A$	$P_{12} = \frac{F_{1A} * ATF_A}{K_{DA} * \left[1 + \frac{ATF_B}{K_{IB}}\right] + ATF_A}$	$\xrightarrow{P_{24}} ITF_B$	$P_{24} = \frac{F_{1B} * ATF_B}{K_{DB} * \left[1 + \frac{ATF_A}{K_{IA}}\right] + ATF_B}$
