# Supplementary material for: Integrating Extrinsic and Intrinsic Cues into a Minimal Model of Lineage Commitment for Hematopoietic Progenitors
Source: PLoS Comput Biol. 2009 Sep 25;5(9):e1000518. doi: 10.1371/journal.pcbi.1000518 (PMC2736398; doi:10.1371/journal.pcbi.1000518)
Supplement: Table S4 — Parameter fitting of microarray data (0.09 MB PDF) [file pcbi.1000518.s006.pdf]

Table S4: Parameter fitting of microarray data

Cell samples collected from FDCP-mix cells that were induced to differentiate along neutrophil, erythrocyte, and megakaryocyte lineages are inherently heterogeneous (Bruno, 2004). To overcome the problem of analyzing aggregate microarray data from these samples, we performed a simple deconvolution by generating a weighting function to account for the non-uniform contribution from each sub-population to the average signal. This is possible since the population fractions at each time point for each sample were previously determined (Bruno, 2004). The weighted parameters (wXi) were obtained from a global fit to the microarray data for all time points across all experiments using a least squares approach and are listed in the table below.

Weighting function:

$$wBi \times fB + wNi \times fN + wEbi \times fEb + wEri \times fEr + wMi \times fM$$

B – blast (CMP)
N – neutrophil
Eb – erythroblast
Er – erythrocyte
M – megakaryocyte

wBi – contribution of blasts to the expression of gene i
fB – fraction of blasts in the population
wNi – contribution of neutrophils to the expression of gene i
fN – fraction of neutrophils in the population
wEbi – contribution of erythroblasts to the expression of gene i
fEb – fraction of erythroblasts in the population
wEri – contribution of erythrocytes to the expression of gene i
fEr – fraction of erythrocytes in the population
wMi – contribution of megakaryocytes to the expression of gene i
fM – fraction of megakaryocytes in the population

Fitted coefficients (wXi) from three independent microarray experiments:

GENE	Blast	Neutrophil	Erythroblast	Erythrocyte	Megakaryocyte
**PU.1**	1.000	4.009	0.379	0.250	0.000
**GATA1**	1.000	0.693	4.233	1.801	1.414
**EPOR**	1.000	0.837	3.021	2.028	2.014
**TPOR**	1.000	0.967	1.679	1.317	4.875
**GCSFR**	1.000	10.425	0.000	0.644	0.000

GENE	Blast	Neutrophil	Erythroblast	Erythrocyte	Megakaryocyte
**PU.1**	1.104	4.071	0.323	0.383	0.000
**GATA1**	1.172	0.754	4.464	2.111	1.545
**EPOR**	1.092	0.906	3.311	2.187	1.979
**TPOR**	1.133	1.240	1.477	1.822	5.662
**GCSFR**	1.071	11.875	0.503	0.377	0.000

GENE	Blast	Neutrophil	Erythroblast	Erythrocyte	Megakaryocyte
**PU.1**	0.896	3.947	0.434	0.118	0.000
**GATA1**	0.828	0.631	4.001	1.491	1.283
**EPOR**	0.908	0.769	2.731	1.869	2.048
**TPOR**	0.867	0.694	1.881	0.812	4.088
**GCSFR**	0.929	8.964	0.000	0.626	0.000

The kinetic expression profile of each gene for each lineage was then obtained by only considering blasts and the cells that are pertinent to the lineage of interest.

For erythrocytes: blasts, erythroblasts and erythrocytes
For megakaryocytes: blasts and megakaryocytes
For neutrophils: blasts and neutrophils

After removing the fractional contribution of cells from other lineages, the remaining populations were normalized to obtain a total fraction of 1.

Reference:

Bruno L, Hoffmann R, McBlane F, Brown J, Gupta R, et al. (2004) Molecular signatures of self-renewal, differentiation, and lineage choice in multipotential hemopoietic progenitor cells *in vitro*. Mol Cell Biol 24: 741-756.
